# High Nuclear Expression of HDGF Correlates with Disease Progression and Poor Prognosis in Human Endometrial Carcinoma

**DOI:** 10.1155/2014/298795

**Published:** 2014-02-17

**Authors:** Lijing Wang, Qingping Jiang, Shengni Hua, Mengyang Zhao, Qiangyun Wu, Qiaofen Fu, Weiyi Fang, Suiqun Guo

**Affiliations:** ^1^Department of Obstetrics and Gynecology, The Third Affiliated Hospital of Southern Medical University, Guangzhou 510630, China; ^2^Cancer Research Institute, Southern Medical University, Guangzhou 510515, China; ^3^Department of Pathology, Third Affiliated Hospital of Guangzhou Medical College, Guangzhou 510150, China

## Abstract

*Aims*. This study examined the correlation between high nuclear expression of hepatoma-derived growth factor (HDGF) and clinicopathologic data in endometrial carcinoma (EC), including patient survival. *Methods*. One hundred and twenty-two endometrial carcinoma (EC) patients from 2002 to 2008 were reviewed in the study. HDGF expression in tumor tissues was examined using immunohistochemistry (IHC), and its association with clinicopathological parameters was evaluated. Tumors with 80% or more nuclei staining were regarded as high expression and tumors with less than 80% nuclei staining considered as low expression. *Results and Conclusions*. Immunohistochemical analysis revealed that HDGF was expressed in both the nucleus and cytoplasm. High nuclear expression of HDGF was positively correlated with FIGO stage (*P* = 0.032), but not associated with other clinical features, such as histological grading or lymph node status. Patients with high expression of HDGF had poorer overall survival rates than those with low expression of HDGF (*P* = 0.001). However, multivariate analyses showed that high nuclear expression of HDGF protein was not an independent predictor of prognosis for EC patients (*P* = 0.111). Our results suggest that high nuclear expression of HDGF is a potential unfavorable factor for the progression and prognosis of EC.

## 1. Introduction

Endometrial carcinoma is the most frequent gynecologic malignancy of women across the globe and the number of estimated new cases shows an increasing trend [1, 2]. If endometrial carcinoma is detected and treated before the cancer has spread outside the uterus, the 5-year relative survival rate is 80% [3]. However, not all endometrial carcinomas can be found at this early stage; thus, metastasis is most often responsible for EC deaths [4].

Hepatoma-derived growth factor (HDGF) was originally purified from HuH-7 liver cancer cell line [5]. Although initially thought to be a cytoplasmic protein, HDGF is a nuclear targeted protein containing a canonical bipartite nuclear localization sequence [6]. Recent studies have found that HDGF expression is increased in several types of mouse and human carcinomas compared with adjacent nontumorous areas [7]. Several findings suggest that HDGF overexpression is associated with aggressive phenotypes of cancer cells, such as proliferation, invasiveness, and metastasis [8–11]. Therefore, HDGF may prove useful as a prognostic factor for patients with cancers.

Thus far, no study has examined the role of HDGF in endometrial carcinoma. This work aimed to study the connections between HDGF expression and the clinicopathologic features including survival, in Chinese patients with EC. We found that patients with high expression of HDGF had poorer overall survival rates than those with low expression of HDGF. Our findings suggest that high nuclear expression of HDGF is a potential unfavorable factor in the progression and prognosis of EC.

## 2. Materials and Methods

### 2.1. Sample Collection

Formalin-fixed and paraffin embedded samples (122) of endometrial carcinoma (EC) (all are endometrioid carcinoma) from 2002 to 2008 were obtained in the Third Affiliated Hospital of Guangzhou Medical School, Guangzhou City, China. All patients with endometrial carcinoma underwent surgery, which consisted of peritoneal cytology, total hysterectomy, bilateral salpingo-oophorectomy, and pelvic and para-aortic lymph node sampling when necessary. No patient experienced chemotherapy or radiotherapy before surgery. Patient ages ranged from 30 to 82 years old. The clinical follow-up time of patients ranged from 48 to 108 months. For the use of these clinical materials for research purposes, prior consent from the patients and approval from the Ethics Committees of this hospital were obtained. All specimens had confirmed pathological diagnosis and were staged according to the FIGO 2009.

### 2.2. Immunohistochemistry

Paraffin sections (3 *μ*m) from 122 EC samples were deparaffinized in 100% xylene and rehydrated in descending ethanol series (100%, 90%, 80%, and 70% ethanol) and water according to standard protocols. Heat-induced antigen retrieval was performed in 10 mM citrate buffer for 2 min at 100°C. Endogenous peroxidase activity and nonspecific antigen were blocked with peroxidase blocking reagent containing 3% hydrogen peroxide and serum, followed by incubation with goat anti-human polyclonal HDGF antibody (1 : 100) (ProteinTech Group, USA) overnight at 4°C. After washing, the sections were incubated with biotin-labeled rabbit anti-goat antibody for 10 min at room temperature and subsequently were incubated with streptavidin-conjugated horseradish peroxidase (HRP) (Maixin Inc, China). The peroxidase reaction was developed by using 3,3 diaminobenzidine chromogen solution in DAB buffer substrate. Sections were visualized with DAB, counterstained with hematoxylin, mounted in neutral gum, and analyzed by using a bright field microscope.

### 2.3. Evaluation of Staining

The immunohistochemically stained tissue sections were reviewed separately by two pathologists blinded to the clinical parameters and evaluated for the presence of nuclear staining. The staining results were defined based on the percentage of positive nuclei staining. Tumors with 80% or more nuclear staining were regarded as high expression, and less than 80% were regarded as low expression.

### 2.4. Statistical Analyses

SPSS 13.0 software was applied to perform all statistical analyses. The *χ* ^2^ test was used to analyze the relationship between the levels of HDGF expression and clinicopathologic characteristics. Survival curves were plotted using the Kaplan-Meier method and compared using the log-rank test. The significance of various variables in survival was analyzed using multivariate cox proportional hazards model. A *P* value of less than 0.05 was considered statistically significant.

## 3. Results

### 3.1. Immunohistochemical Analysis of HDGF Protein Expression in EC Tissues

**Figure 1:**
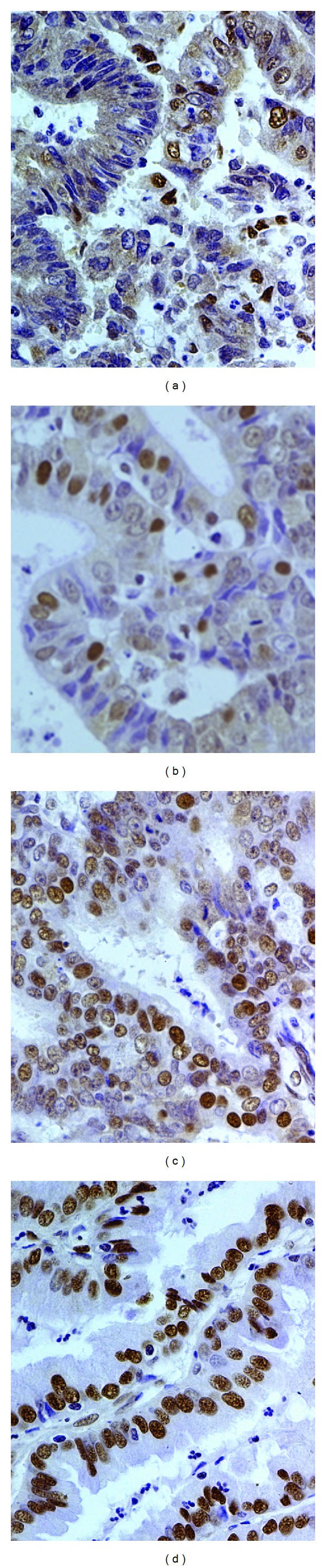
HDGF protein is expressed in the nuclei of malignant epithelial cells for EC samples (original magnification: ×400). (a)–(d) HDGF protein expression in cellular nucleus of EC tissues; (a)-(b) low expression; (c)-(d) high expression.

We measured expression levels and subcellular localization of HDGF protein in 122 archived paraffin-embedded EC samples using immunohistochemical staining (Figure 1). Specific HDGF protein staining was detected in the nuclei and cytoplasm of noncancerous and malignant epithelial cells but was more pronounced in the nucleus. We observed that 25.5% (31/122) and 74.5% (91/122) (Table 1) of cases exhibited high and low nuclear expression of HDGF, respectively.

### 3.2. Relationship between Clinicopathological Characteristics and HDGF Nuclear Expression Level in EC Patients

Based on the significance of nuclear HDGF expression in previous studies of tumors [12], we investigated the correlation of nuclear HDGF expression with clinical features and prognosis of EC. As shown in Table 1, we did not find a significant association between HDGF nuclear expression and patient's age, menopausal status, histological grading, depth of myometrial invasion, or lymph node status in 122 EC cases. However, we observed that high nuclear expression of HDGF was positively correlated with FIGO stage (I-II versus III) (*P* = 0.032) in EC patients (Table 1).

### 3.3. HDGF High Expression Is Associated with Overall Survival Time of EC

**Figure 2:**
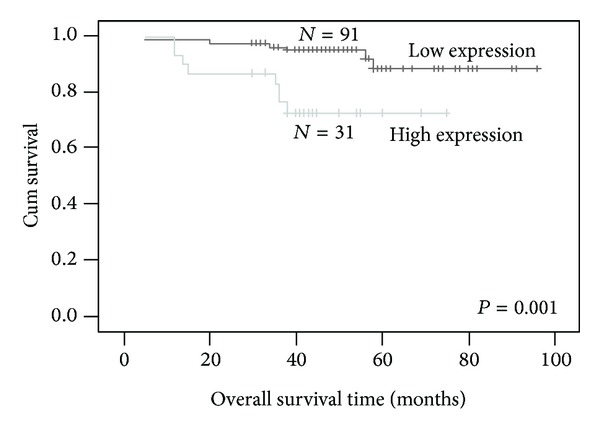
Nuclear expression of HDGF protein predicts EC patients' overall survival time. Patients with HDGF high expression had worse survival than those with low expression of HDGF (*P* = 0.001).

To investigate the prognostic value of HDGF expression for EC, we assessed the association between the levels of HDGF expression and patient survival using Kaplan-Meier analysis with the log-rank test. In 122 EC cases with prognosis information, we observed that the level of HDGF nuclear protein expression was significantly correlated with overall survival. Patients with high expression had worse prognoses than those with low expression of HDGF (Figure 2) (*P* = 0.001).

### 3.4. High HDGF Expression Is Inversely Associated with Survival Time of EC Patients Based on Depth of Myometrial Invasion (≧1/2), Lymph Node Metastasis, without Lymph Node Metastasis and FIGO Stage III

**Figure 3:**
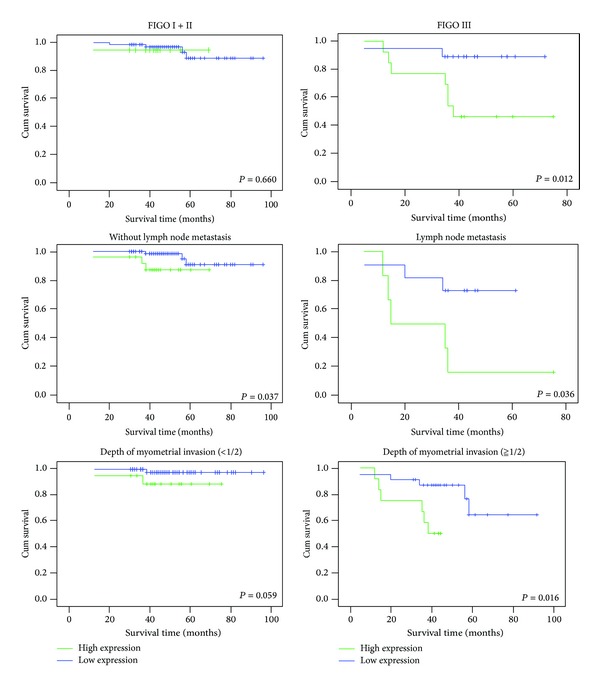
The correlation of HDGF expression with EC patients' survival time in strata analysis against depth of myometrial invasion, lymph node metastasis, and FIGO stage. HDGF protein expression was significantly associated with the survival time for EC patients in FIGO stage III (*P* = 0.012), lymph node metastasis (*P* = 0.036), without lymph node metastasis (*P* = 0.037), and depth of myometrial invasion (≧1/2) (*P* = 0.016) but did not correlate with FIGO stage I + II (*P* = 0.66) or depth of myometrial invasion (<1/2) (*P* = 0.059).

We further analyzed the correlation between HDGF high expression and prognosis for EC patients by strata analysis against lymph node status, depth of myometrial invasion, and FIGO stage. These results indicated that high HDGF protein expression was significantly associated with the survival time for EC patients based on depth of myometrial invasion (≧1/2) (*P* = 0.016), lymph node metastasis (*P* = 0.036), without lymph node metastasis (*P* = 0.037) and FIGO stage III (*P* = 0.012) (Figure 3).

### 3.5. High Nuclear Expression of HDGF Is Not an Independent Prognosis Factor for EC Patients

Univariate analyses showed that FIGO stage, histological grading, lymph node status, depth of myometrial invasion, postoperative hormone therapy, and high HDGF nuclear expression were also significantly correlated with patients' survival (*P* = 0.002, *P* = 0.001, *P* < 0.001, *P* < 0.001, *P* = 0.044, and *P* = 0.004, resp.). To determine whether HDGF is an independent prognostic factor for EC, we performed multivariate analysis of HDGF protein expression levels adjusted for FIGO stage, histological grading, lymph node status, depth of myometrial invasion, and postoperative hormone therapy of EC patients. These results showed that the level of HDGF expression was not an independent prognostic factor for EC (*P* = 0.111) (Table 2).

## 4. Discussion

Similar to the well-established adenoma-to-carcinoma progression of colon cancer development, sporadic EC is believed to develop through a continuum from complex atypical hyperplasia (CAH) to well-differentiated cancer. Risk factors for sporadic endometrioid EC include age, obesity, nulliparity, and an excess of estrogen to progesterone ratio [13–15]. Chronic estrogen exposure is believed to trigger the transformation to hyperplasia followed by EC [16]. However, the molecular mechanisms underlying the pathogenesis of EC remain incompletely understood.

HDGF is known to play an important role in vascular growth and formation by regulating endothelial cell proliferation and migration [17, 18]. Thus, HDGF derived from tumors may promote tumor growth and angiogenesis via paracrine mechanisms.

Recent reports have shown that high nuclear expression of HDGF facilitates the progression and poor prognosis in some primary cancers, including hepatocellular [19], gastric [20], pancreatic [21], non-small-cell lung cancers [22], and gastrointestinal stromal tumors [23]. However, the correlation between nuclear HDGF expression and clinical features has not been reported in EC.

In this study, we found that although high nuclear expression of HDGF level was not associated with most clinical features, such as depth of myometrial invasion and lymph node status, it was positively correlated with FIGO stage. This hints that high nuclear expression of HDGF promotes the clinical progression of EC and further supports the role of HDGF as a potential oncogene in tumors.

Recently, high nuclear expression of HDGF was shown to be an unfavorable independent prognostic factor in some types of tumors [19–23]. In this investigation, we also presented evidence that high nuclear expression of HDGF protein in EC was inversely correlated with patient's overall survival time. Patients with high nuclear expression of HDGF protein had a shorter overall survival time. Our result suggests that high nuclear expression of HDGF is a clinically relevant biomarker for EC prognosis.

It is well known that invasion and metastasis of tumor cells are the key causes of death for EC patients. We assessed survival prognosis by strata analysis against clinical features associated with invasion and metastasis including lymph node metastasis status, depth of myometrial invasion, and FIGO stage. Interestingly, we observed that nuclear expression of HDGF protein was significantly associated with the survival time for EC patients in depth of myometrial invasion (≧1/2), lymph node metastasis, without lymph node metastasis, and FIGO stage III. Patients with high nuclear expression of HDGF protein had a shorter overall survival time in these groups. We speculate that the results are correlated with HDGF function in these tumors. In previous studies, HDGF had been reported as an important tumor metastasis promoter by facilitating epithelial-mesenchymal transition and cell metastasis [24]. When patients are in later stages of EC, tumor cells can not be completely eliminated by standard therapy means such as surgical operation or chemotherapy. Therefore, residual cells with higher expression of HDGF exert a more powerful stimulation for invasion and metastasis, which may promote the death of more patients in EC.

Finally, we analyzed possible independent prognosis factors correlated with the pathogenesis of EC including nuclear expression of HDGF. Although FIGO stage, histological grading, lymph node status, depth of myometrial invasion, postoperative hormone therapy, and nuclear HDGF expression were statistically significant based on univariate analysis, none were independent prognosis factors for EC pathogenesis, we speculate that this might be attributed to the superior survival rates for EC patients [25]. Among 122 patients that we studied within the clinical follow-up time, only 23 patients died. Limited sample size might have also contributed to these results. Nonetheless, these key clinical parameters remain statistically significant independent prognosis factors for EC.

## 5. Conclusion

In summary, we have shown for the first time that high nuclear expression of HDGF may be involved in the clinical progression and poor prognosis of EC patients. Furthermore, our results suggest that nuclear expression of HDGF may be a new clinically significant biomarker for EC prognosis. Due to the limited sample size of patients in our study, further investigations are needed to confirm these findings and establish the role of HDGF as a reliable clinical predictor for endometrial carcinoma outcome. Our findings suggest that inhibition of HDGF activation could be an effective approach for slowing the disease and provide a basis for the application of HDGF inhibitors in the future.

## Figures and Tables

**Table 1:** Correlation between the clinicopathologic characteristics and nuclear expression of HDGF protein in EC.

HDGF (%)				
Characteristics	*N*	High expression	Low expression	*P*
Age				
<50	42	10 (23.8%)	32 (76.2%)	0.830
≧50	80	21 (26.3%)	59 (73.7%)	
Menopausal status				
Premenopausal	65	15 (23.1%)	50 (76.9%)	0.537
Postmenopausal	57	16 (28.1%)	41 (71.9%)	
FIGO stage				
I-II	90	18 (20%)	72 (80%)	0.032
III	32	13 (40.6%)	19 (59.4%)	
Histological grading				
G1	44	15 (34.1%)	29 (65.9%)	0.140
G2	62	11 (17.7%)	51 (82.3%)	
G3	16	5 (31.3%)	11 (68.7%)	
Depth of myometrial invasion				
<50%	85	19 (22.4%)	66 (77.6%)	0.263
≧50%	37	12 (32.4%)	25 (67.6%)	
Lymph node status				
Negative	105	25 (23.8%)	80 (76.2%)	0.369
Positive	17	6 (35.3%)	11 (64.7%)	

**Table 2:** Summary of univariate and multivariate Cox regression analysis of overall survival duration.

Parameter	Univariate analysis			Multivariate analysis		
	*P*	HR	95% CI	*P*	HR	95% CI
Age						
<50 versus ≧50	0.093	0.401	0.138–1.165			
Family history of tumor						
Negative versus positive	0.279	0.325	0.043–2.487			
Education						
<Graduation versus ≧graduation	0.298	26.921	0.055–13271.753			
Health Insurance						
No versus yes	0.089	0.020	0.000–1.811			
Career						
≦Worker versus >worker	0.272	27.978	0.073–10713.674			
Menopausal status						
Premenopausal versus postmenopausal	0.559	0.721	0.240–2.160			
Complications						
With versus without	0.125	0.309	0.069–1.384			
FIGO stage						
I + II versus III	0.002	5.652	1.892–16.883	0.729	1.392	0.215–9.006
Histological grading						
G1 versus G2 versus G3	0.001	4.514	1.896–10.745	0.097	2.170	0.870–5.412
Lymph node status						
Negative versus positive	≤0.001	12.232	4.196–35.659	0.070	5.543	0.872–35.231
Depth of myometrial invasion						
<50% versus ≧50%	≤0.001	9.745	2.713–34.999	0.090	3.673	0.818–16.498
HDGF expression						
Low expression versus high expression	0.004	4.951	1.686–14.544	0.111	2.828	0.787–10.165
Postoperative irradiation						
Yes versus no	0.512	1.652	0.368–7.409			
Postoperative chemotherapy						
Yes versus no	0.175	2.081	0.721–6.005			
Postoperative hormone therapy						
Yes versus no	0.044	0.267	0.074–0.963	0.703	0.748	0.169–3.322

## Abbreviations

HDGF: Hepatoma-derived growth factor
EC: Endometrial carcinoma
FIGO: Federation International of Gynecology and Obstetrics.
